# Complete Genome Sequence of an H12N8 Avian Influenza Virus Isolated from Wild Bird Feces in Hunan East Dongting Lake National Nature Reserve

**DOI:** 10.1128/genomeA.00891-13

**Published:** 2013-10-24

**Authors:** Hong Zhang, Zi Li, Hao Yang, Yunzhi Liu, Fangcai Li, Lijie Wang, Xiaodan Li, Yun Zhu, Yahui Cai, Zhiyong Bai, Feiyue Yi, Yuelong Shu

**Affiliations:** National Institute for Viral Disease Control and Prevention, China CDC, Key Laboratory for Medical Virology, National Health and Family Planning Commission, Beijing, Chinaa; Hunan Provincial Center for Disease Control and Prevention, Changsha, Chinab; Yueyang Center for Disease Control and Prevention, Yueyang, Chinac; Hunan East Dongting Lake National Nature Reserve, Yueyang, Chinad

## Abstract

An H12N8 subtype avian influenza virus (AIV) was isolated from a wild bird in China in 2011. It is the first report of isolation of the H12N8 subtype AIV in Asia. Phylogenetic analysis results suggested it is a reassortant, and all eight gene segments belong to the Eurasian gene pool.

## GENOME ANNOUNCEMENT

Wild aquatic birds are natural reservoirs of all subtypes of avian influenza viruses (AIVs) (1–3). Long-term surveillance of AIVs in wild aquatic birds in North America and Europe has occurred since 1976 and 1998, respectively (4, 5). However, little systematic surveillance of AIVs has been performed in wild waterfowl in mainland China. In order to understand the ecology and evolution of AIVs in wild birds in central China, we conducted a continuous survey from November 2011 to April 2012 in the Hunan East Dongting Lake National Nature Reserve, Yueyang, Hunan, China, an important overwintering habitat of East Asian migratory birds, located at the middle reaches of the Yangtze River. Around the lake, there are a number of domestic birds, especially ducks and geese, which can share the water area. This may provide opportunities for cross-species transmission of AIVs. Therefore, continuous surveillance in these areas is very important.

We isolated an H12N8 subtype AIV from wild aquatic bird feces in November 2011 and named the strain A/wild goose/Dongting/C1037/2011 (H12N8), which is the first report of the detection of an H12N8 subtype AIV in Asia. The eight gene segments were amplified and sequenced by the ABI 3730xl DNA analyzer. The complete viral genome consists of 8 gene segments of negative-sense, single-stranded RNA, and it encodes 11 proteins with the amino acid lengths of 759 for polymerase basic 2 (PB2), 727 for PB1, 80 for PB1-F2, 716 for polymerase (PA), 564 for hemagglutinin (HA), 498 for nucleoprotein (NP), 470 for neuraminidase (NA), 252 for M1, 97 for M2, 225 for nonstructural 1 (NS1), and 121 for NS2 proteins. The amino acid sequence of the HA cleavage site is VPQAQDR↓GLF, representing low pathogenicity in chickens (6). The HA protein of the strain maintained the avian-like motif Q^226^ and G^228^ (H3 numbering) at the receptor binding sites (7). An analysis of potential glycosylation site motifs N-X-S/T revealed eight sites at positions 28, 140, 151, 222, 302, 309, 496, and 523 in HA and seven sites at positions 46, 54, 67, 84, 144, 293, and 398 in NA. The isolate possesses E and D at positions 627 and 701 of the PB2 protein, which are characteristic of avian influenza viruses (8, 9).

A phylogenetic analysis of the isolate showed that all eight segments belong to the Eurasian lineage. Also, its internal genes are closely related to H7N7, H10N1, H5N2, H10N2, H11N2, and H6N5 subtype AIVs, which suggests that the virus underwent extensive reassortment with different subtype influenza viruses circulating around the Eurasian continent.

In conclusion, the isolate is a novel reassortant virus genotype, and the complete sequence of the strain is the first full-genome data of an H12N8 subtype AIV isolated from Eurasia. Our results highlight the necessity of constant surveillance of AIVs in this region to better understand the ecology and evolution of the AIVs in wild aquatic birds.

### Nucleotide sequence accession numbers.

The genome sequences of A/wild goose/Dongting/C1037/2011 (H12N8) have been deposited in GenBank under accession no. KC876688 to KC876695.
